# PMI estimation through ^1^H NMR metabolomics on human pericardial fluid: a validation study

**DOI:** 10.1007/s11306-025-02376-3

**Published:** 2025-11-15

**Authors:** Alberto Chighine, Matteo Stocchero, Fabio De-Giorgio, Riccardo Fratini, Giorgia Fanunza, Radhika Kesharwani, Camilla Gozzelino, Matteo Nioi, Ernesto d’Aloja, Emanuela Locci

**Affiliations:** 1 Department of Medical Sciences and Public Health, Section of Legal Medicine, University of Cagliari, Cagliari, CA Italy; 2 Department of Women’s and Children’s Health, University of Padova, Padova, Italy; 3 Department of Health Surveillance and Bioethics, Section of Legal Medicine, Catholic University of Rome, Rome, Italy; 4 Fondazione Policlinico Universitario A. Gemelli IRCCS, Rome, Italy

**Keywords:** Human pericardial fluid, ^1^H NMR metabolomics, PMI, Extraction protocol, Time since death, Post-mortem

## Abstract

This study builds on a prior proof-of-concept metabolomic analysis of post-mortem pericardial fluid to assess its reproducibility and validate its utility for estimating the post-mortem interval. Sixty-five pericardial fluid samples were collected during medico-legal autopsies in two different Forensic Medicine Institutes with post-mortem intervals spanning 16 to 199 h. Samples underwent liquid-liquid extraction and ^1^H NMR analysis, quantifying 50 metabolites. Multivariate statistical analyses were employed to develop post-mortem interval estimation models, controlling for age to minimize its confounding effects. Reproducibility was confirmed, with 92% of metabolites showing high similarity (cosine similarity ≥ 0.90) in 23 re-analyzed samples, demonstrating robust intra-laboratory consistency. For post-mortem intervals of 16 to 100 h, the regression model achieved presented a prediction error of 16.7 h, identifying nine key predictors, including choline, glycine, citrate, betaine, ethanolamine, glutamate, ornithine, uracil, and β-alanine. For intervals of 16 to 130 h, the prediction error was 23.2 h, and for 16 to 199 h, it was 42.1 h. A classification model distinguishing intervals below 48 h from those above 48 h showed high accuracy for detecting longer intervals, with key predictors including aspartate, histidine, and proline. These findings underscore the stability and reproducibility of pericardial fluid metabolomics, establishing its potential as a reliable forensic tool for post-mortem interval estimation, particularly beyond 48 h, with significant implications for forensic investigations.

**Supplementary Information:**

The online version contains supplementary material available at 10.1007/s11306-025-02376-3.

## Introduction

Pericardial fluid (PF) is a serous fluid found in the pericardial cavity. It is a product of plasma ultrafiltration but also arises from secretions by pericardial mesothelial cells and myocardial interstitial fluid. This suggests that PF plays a role in pericardial and cardiac homeostasis, containing bioactive molecules with potential paracrine functions (Trindade et al., 2019).

Due to ethical constraints, PF cannot be collected from healthy individuals, and samples are typically obtained during open-heart surgeries, where the fluid’s composition may be altered by underlying conditions like valvular or ischemic heart diseases (Fatehi Hassanabad et al., 2021). Only few recent studies have explored PF’s chemical and biochemical composition (Buoro et al., 2021; Imazio et al., 2020; Ben-Horin et al., 2005), while omics approaches have been limited to a lipidomic investigation in a rabbit model of hypercholesterolemia (Tamura et al., 2018) and a metabolomic study in living patients undergoing cardiac surgery for coronary artery bypass grafting (Yang et al., 2023).

In the post-mortem settings, the use of PF has primarily focused on determining the cause of death and conducting toxicological analysis (Kawamoto et al., 2013) as presented in the latest studies based on the use of post-mortem PF (Takasu et al., 2025; Martínez-Jiménez et al., 2025; Minakata et al., 2024; Wu et al., 2024; Kim et al., 2024). Very recently, we introduced the first post-mortem metabolomic study on human PF with the aim of investigating the correlation between PF metabolomic profiles and the post-mortem interval (PMI) (Chighine et al., 2023), showing the feasibility of obtaining a calibration model for PMI estimation. In the present paper, we aim to evaluate the reproducibility of our preliminary data and its validation on a larger dataset.

## Materials and methods

### Sample collection

PF samples (66) were collected during medico-legal autopsies performed at Forensic and Legal Medicine Institute of University of Cagliari (*n* = 43) and of Catholic University of Rome (*n* = 23). 23 out of the 43 samples gathered in Cagliari were the same used in the previous proof-of-concept study (Chighine et al., 2023).

Following the standard procedure to open the chest wall, the pericardial cavity was carefully exposed using an inverted ‘Y’ incision. The heart’s apex was then delicately lifted, and the declivous PF was gathered using a sterile no-needled syringe. No collection took place if there was evident pathological pericardial effusion or blood contamination by visual inspection.

For each individual, data on sex, age, cause of death, and PMI were recorded. However, not all deaths were eye-witnessed, as some PMIs were estimated by circumstantial evidence (i.e., preliminary police investigations findings) or traditional thanatological signs, namely *algor*, *livor*, and *rigor mortis*. Once collected, PF samples were immediately frozen at − 80 °C. Samples obtained in Rome were shipped on dry ice to the Forensic and Legal Medicine Institute of University of Cagliari.

Since all samples were gathered during medico-legal autopsies, informed consent was secured via the local Prosecutor’s office, which acted in what was considered the best interest of the deceased, with all samples fully anonymized.

### Sample preparation for NMR analysis

PF samples were prepared for NMR analysis using a liquid-liquid extraction (LLE) procedure as described in the previous work (Chighine et al., 2023). The 23 PF samples consistent with the proof-of-concept dataset underwent *ex novo* independent LLE extraction and NMR analysis along with the 42 samples of the new dataset.

### ^1^H NMR experiments and data processing

^1^H NMR experiments were carried out on a Varian UNITY INOVA 500 spectrometer (Agilent Technologies, CA, USA) operating at 499.839 MHz. The spectra were acquired using the previously reported experimental conditions and spectral processing to enable the comparison with the previous dataset. Indeed, using the Chenomx NMR Suite Profiler tool (Chenomx Inc., Edmonton, Canada), the same set of 50 metabolites was quantified, after the exclusion of exogenous metabolites such as ethanol, caffeine, and drugs (see Supplementary Table 1). The final data set was exported as a text file for multivariate statistical data analysis. Data were autoscaled prior to performing data analysis.

### Multivariate statistical data analysis - NMR data

The similarity between the concentration of the metabolites in the previous proof-of-concept and new extended datasets was assessed calculating the cosine similarity. Exploratory data analysis was performed by Principal Component Analysis (PCA), aiming to detect outliers and specific patterns within the data (Joliffe, 2002). A training set was extracted from the collected samples as follows. The PMI range was segmented into subsets. For each subset, PCA and Hierarchical Cluster Analysis based on the Ward’s method were applied to determine a number of clusters equal to the 70% of the number of samples of that subset. The samples closest to the center of the clusters were included in the training set, while the remaining samples were assigned to the test set. Regression models for PMI estimation based on the quantified metabolites were developed using orthogonally Constrained PLS2 (oCPLS2) (Stocchero et al., 2018). Implementing orthogonal constraints in PLS-regression was essential to avoid the influence of age on the models. Specifically, oCPLS2 ensured that score components remained independent of age, focusing the modeling on data variations primarily linked to PMI. Model optimization was conducted using 20-repeated 5-fold cross-validation, aiming to maximize the R² value on the validation set, i.e. Q², under the condition to pass the randomization test on the response (1000 random permutations). Key predictors were identified through stability selection, utilizing the Variable Influence on Projection (VIP) score for variable selection (Stocchero, 2020). Moreover, Multiple Linear Regression (MLR), considering age and PMI as independent variables, was applied.

In the case of the comparison of two different classes, orthogonally constrained PLS for classification (oCPLS2C) (Stocchero et al., 2021) with stability selection based on VIP was applied (Stocchero, 2020). The model was constrained by age. The number of PLS-score components was assessed maximizing the Matthews correlation coefficient (MCC) calculated by 10-repeated 5-fold full cross-validation under the condition to pass the randomization test on the class (1000 random permutations). Moreover, logistic regression was applied to investigate the effect of age and of the metabolite concentration on the two classes.

In stability selection, 200 subsets were generated applying Binary Matrix Sampling with a probability of 0.7 to both observations and predictors. For each subset, the group of predictors yielding the PLS model with the best-performance in cross-validation was determined by feature selection based on VIP. The selection frequency of each predictor was then compared to that of a random selection to pinpoint the most significant predictors. A significance level of 0.05 was assumed.

PCA was performed by Simca 14 (Umetrics, Sweden), whereas oCPLS2, oCPLS2C, stability selection, MLR-based analysis, logistic analysis and sample selection were implemented by in-house R-functions developed using R 4.2.2 (R Foundation for Statistical Computing, Vienna).

## Results

A total of 65 PF samples, gathered from medico-legal autopsies conducted at two distinct Legal Medicine Institutes, composed the final extended dataset. Of these, 23 PF samples were the same used in the previous proof-of-concept experiments (Chighine et al., 2023), excluding sample #6 which was depleted during the initial study. Based on the proof-of-concept findings, the LLE method was selected for removing macromolecules prior to ^1^H NMR analysis. This choice was driven by the fact that ultrafiltration and LLE procedures yielded to highly comparable metabolomic profiles, LLE giving better accuracy in PMI prediction and allowing to retain the lipophilic phase for further analysis on complementary analytical platforms.

Briefly, PF samples were collected from deceased individuals of both sexes (M to F ratio of roughly 2:1), aged ranging 18 to 87 years (mean = 52.2, SD = 20.5), and with PMI spanning from 16 to 199 h (mean = 77.4, SD = 42.7) – see Table 1.

**Table 1:** Demographic details of the studied cases, including post-mortem interval (PMI), cause of death, and the location where autopsies were performed (CA = Cagliari, RM = Rome). Samples 1 to 24 were used in the proof-of-concept experiment. All samples except #6 were included in the extended dataset experiment (*n* = 65 individuals)

#	PMI	Sex	Age	Cause of Death	Autopsy site	#	PMI	Sex	Age	Cause of Death	Autopsy site
1	16	F	31	Haemorragic Shock	CA	34	69,5	M	37	Acute Cardiac Failure	CA
2	20	M	20	Mechanical Asphyxia (Hanging)	CA	35	85	M	73	Haemorragic Shock in Aneurismatic Rupture	CA
3	25	M	35	Acute Cardiac Failure	CA	36	96	M	48	Traumatic Shock	CA
4	32	M	21	Epidural Hematoma	CA	37	48	M	30	Mechanical Asphyxia (Aspiration)	CA
5	33	M	87	Subdural Hematoma	CA	38	110	F	35	Septic Shock in Intestinal Perforation	CA
6	33.5	F	52	Cardiogenic Shock (Myocardial Infarction)	CA	39	93	F	66	Acute Cardiac Failure	CA
7	38	M	68	Haemorrhagic and Traumatic Shock	CA	40	47	F	29	Acute Cardiac Failure	CA
8	39	M	63	Mechanical Asphyxia (Positional)	CA	41	78	F	41	Traumatic Shock	CA
9	41	F	81	Acute Cardiac Failure	CA	42	53	M	52	Traumatic Shock	CA
10	46	M	32	Drug Intoxication (Cocaine)	CA	43	53	M	35	Traumatic Shock	CA
11	46	M	38	Mechanical Asphyxia (Hanging)	CA	44	75	F	45	Traumatic Shock	RM
12	62	F	60	Pulmonary Embolism and Cerebellar Ischemia	CA	45	141	M	62	Traumatic Shock	RM
13	63	M	42	Cerebral Ischemia	CA	46	63	M	59	Acute Cardiac Failure	RM
14	66	M	37	Haemorragic Shock	CA	47	130	F	55	Multiorgan Failure in Renal Carcinoma	RM
15	70,3	M	49	Mechanical Asphyxia (Aspiration)	CA	48	74	F	78	Traumatic Shock	RM
16	77	F	39	Multiorgan Failure	CA	49	170	F	74	Traumatic Shock	RM
17	82	M	28	Drug Intoxication (Cocaine, Heroin)	CA	50	60	M	53	Mechanical Asphyxia (Hanging)	RM
18	86	M	58	Acute Bowel Infarction	CA	51	39	M	48	Acute Cardiac Failure	RM
19	92	M	32	Hypoxic Brain Death	CA	52	199	M	44	Multiorgan Failure in COVID-19	RM
20	100	M	81	Haemorrhagic Shock	CA	53	36	M	23	Traumatic Shock	RM
21	120	F	77	Neoplastic Cachexia	CA	54	54	F	39	Acute Cardiac Failure	RM
22	160	M	42	Drug Intoxication (Buprenorphine)	CA	55	125	F	85	Acute Cardiac Failure	RM
23	168	F	69	Neoplastic Cachexia	CA	56	31	F	24	Acute Cardiac Failure	RM
24	170	M	80	Acute Cardiac Failure	CA	57	42	F	45	Traumatic Shock	RM
25	106	M	87	Traumatic Shock	CA	58	154	M	44	Drug Intoxication (Cocaine, Heroin)	RM
26	55,5	M	28	Acute Cardiac Failure	CA	59	73,5	M	82	Mechanical Asphyxia (Hanging)	RM
27	70	F	71	Acute Cardiac Failure	CA	60	97	F	57	Caustic Aspiration	RM
28	47	M	15	Traumatic Brain Injury	CA	61	105	M	81	Acute Cardiac Failure	RM
29	54	M	20	Haemorrhagic Shock	CA	62	70,5	M	65	Acute Cardiac Failure	RM
30	45	M	72	Acute Cardiac Failure	CA	63	168	M	75	Acute Cardiac Failure	RM
31	104	M	81	Septic Shock	CA	64	26,5	F	54	Mechanical Asphyxia (Hanging)	RM
32	64	M	59	Traumatic Brain Injury	CA	65	73	M	51	Mechanical Asphyxia (Hanging)	RM
33	65	M	21	Traumatic Shock	CA	66	32	M	80	Acute Cardiac Failure	RM

### Reproducibility assessment with the proof-of-concept experiment

To evaluate the intra-laboratory reproducibility of the PF ^1^H NMR metabolomic results, a group of 23 PF samples, previously investigated in the proof-of-concept experiment were independently re-processed and re-examined.

**Fig. 1:**
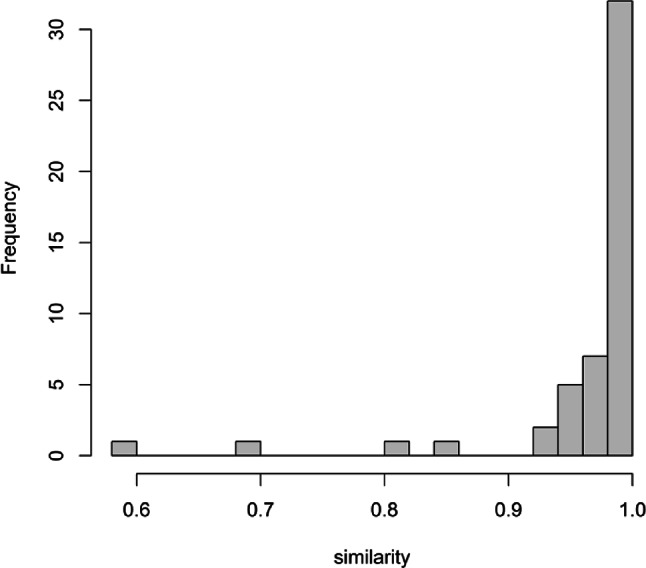
Cosine similarity between the 50 metabolites from the two batches

The similarity between these two separate batches of the same 23 PF samples was initially evaluated by comparing their quantified metabolomes. On the basis of the cosine similarity between the 50 metabolites, 46 exhibited strong similarity (more than 0.90), while the remaining four – Mannose, Maltose, Methanol, and Creatinine – displayed a lower similarity (see Fig. 1).

Given the complex nature of the experimental protocol, which encompasses multiple stages – from the extraction process to the pre- and post-processing of spectral data – this result reflects a remarkably high reproducibility of the overall experiment. The vast majority of the metabolome (92%) showed consistency across the two batches. The lower similarity observed for the four metabolites might be attributed to specific factors. For Mannose and Maltose, their multiple NMR signals, distributed across the aliphatic region of the spectra, may have been deconvoluted differently. Conversely, Methanol and Creatinine, could have been misassigned due to minor shifts in their chemical positions.

To facilitate comparison, the oCPLS2 results of the proof-of-concept experiment were constrained to the 23 shared PF samples. The division into training and test sets mirrored that of the proof-of-concept experiment - with the exception of sample #6 - resulting in a training set of 17 samples and a test set of 6 samples. When the full 170-hour time window was analyzed, the new dataset exhibited moderate consistency, with increased standard deviation error in prediction (SDEP − 41.0 vs. 34.2 h), even after excluding the four metabolites with lower similarity (42.1 vs. 39.5 h). However, when PMI exceeding 100 h were excluded, reproducibility improved significantly, yielding a SDEP of 10.5 and 12.0 h for the old and new batch, respectively. Notably, the 24 samples proof-of-concept experiment reported a SDEP of 13.0 h. When only the selected predictors were considered, prediction accuracy showed a slight enhancement, with SDEPs of 10.1 and 11.7 h for the old and new batch, respectively. The results are listed in Table 2.

**Table 2:** oCPLS2 prediction errors of models based on previous (Chighine et al., 2023) and extended dataset validated with a test set of 6 independent samples. (*) selected metabolites excluding Maltose, Mannose, Methanol, creatinine

PMI range (h)	training set (samples)	SDEP (hours)
16–170	Proof-of-concept dataset (*n* = 18)	34.0
	Proof-of-concept dataset (*n* = 17)	34.2
	Extended dataset (*n* = 17)	41.0
	Proof-of-concept dataset (*n* = 17) *	39.5
	Extended dataset (*n* = 17) *	42.1
16–100	Proof-of-concept dataset (*n* = 18)	13.0
	Proof-of-concept dataset (*n* = 17)	10.5
	Extended dataset (*n* = 17)	12.0
	Proof-of-concept dataset (*n* = 17) *	10.1
	Extended dataset (*n* = 17) *	11.7

### Autopsy site and/or sample shipment effect

**Fig. 2:**
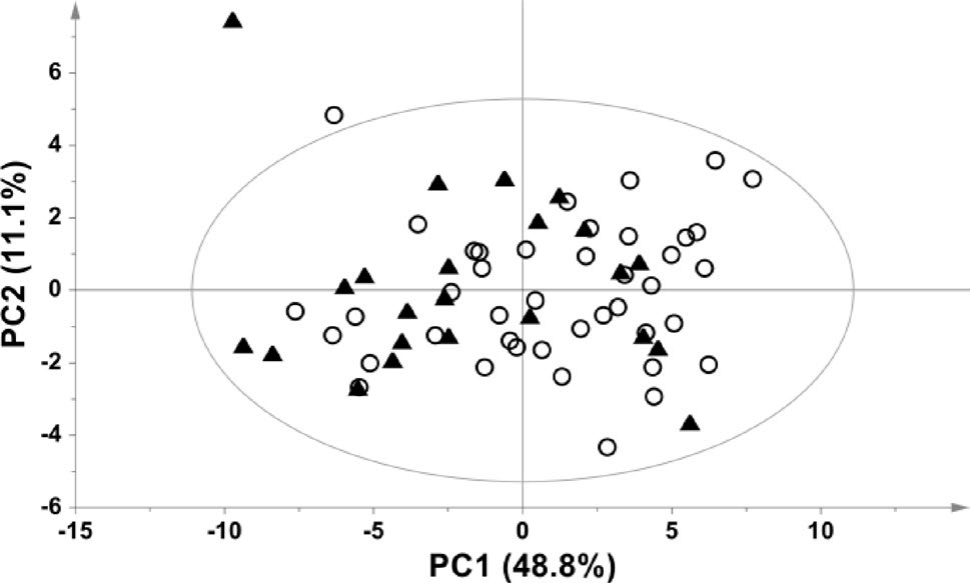
PCA model of all the collected PF samples. Samples are identified according to the sampling site (CA = Cagliari, open circles; RM = Rome, black triangles)

An exploratory PCA was conducted to investigate potential influences from the autopsy location or sample shipment. The PCA score plot shows that the samples are randomly distributed within the multivariate space independently on their collection site, suggesting that neither the geographical differences nor the variation in operators performing the sampling impacts the PF metabolomic composition (Fig. 2). Additionally, this result confirms that dry ice effectively preserves samples integrity during transportation.

### Regression model on the PMI window 16–199 h

Compared to the proof-of-concept study, the new PF dataset was expanded to include 65 samples, encompassing PMIs up to 199 h. As previously, a moderate correlation between PMI and age was observed (*r* = 0.35, *p* = 0.02), while no significant correlation was detected between PMI and sex. Supervised data analysis based on Projection to Latent Structure regression (PLS) was applied to evaluate the effects of PMI on the metabolomic profiles of the collected PF samples. The new dataset was split into a training set of 45 samples, used to construct the model, and an independent test set of 20 samples, with both sets balanced for PMI, sex and age distributions.

**Fig. 3:**
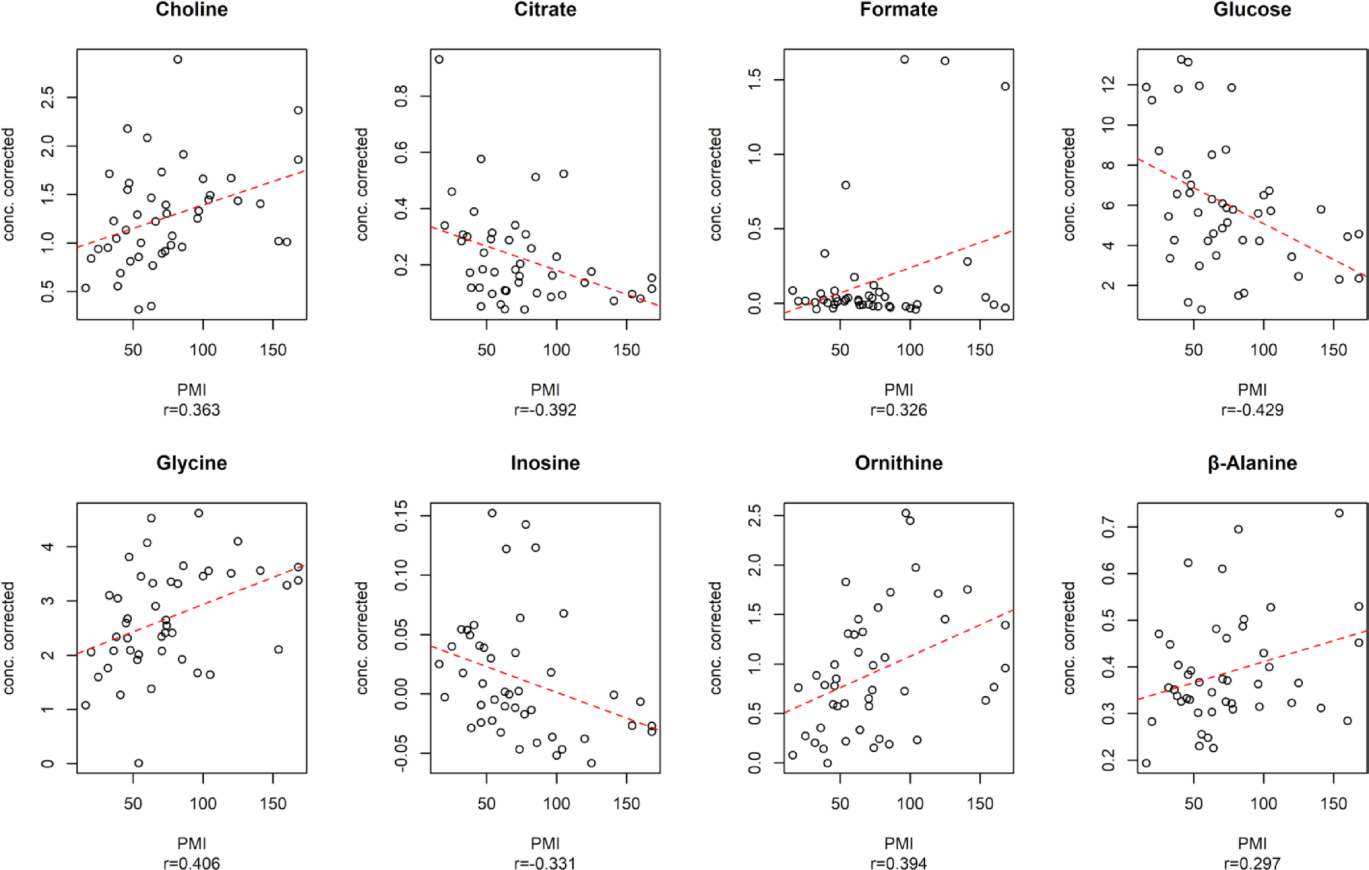
Relevant predictors for oCPLS2 model in 16–199 h PMI window. The dashed red line indicates the linear regression between the metabolite concentration corrected by age and the experimental PMI; r is the Pearson correlation coefficient

The oCPLS2 model, incorporating stability selection and relying solely on eight relevant predictors, showed 1 predictive score component, R^2^ = 0.256 (*p* = 0.019), Q^2^ = 0.163 (*p* = 0.001), standard deviation error in calculation (SDEC) of 33.5 h, mean standard deviation error in cross-validation (SDECV) of 35.6 h and and standard deviation error in prediction (SDEP) of 42.1 h. Figure 3 highlights the eight relevant predictors identified, five of which showed a positive correlation with PMI (Choline, Formate, Glycine, Ornithine, and β-Alanine) while the other three decreased as PMI increased (Citrate, Glucose, Inosine).

The regression model maintained comparable performance in prediction, even with the significantly extended PMI range examined and the larger number of samples included.

### Regression model on the PMI window 16–130 h

Given that medico-legal autopsies are seldom conducted beyond five or six days after death, PMIs greater than 130 h were again underrepresented as in the proof-of-concept experiment (comprising only 8 of the 65 samples). As a result, a new model was developed, targeting a PMI range of 16 to 130 h and utilizing a dataset of 57 samples. A moderate correlation between PMI and age was again observed (*r* = 0.37, *p* = 0.02) while no association with sex emerged. The samples were divided into a training set of 40 samples and an independent test set of 17 samples both balanced for PMI, sex and age distributions.

The oCPLS2 model, refined with stability selection and based on nine relevant predictors, identified 1 predictive score component yielding an R^2^ of 0.328 (*p* = 0.002) and a Q^2^ of 0.217 (*p* = 0.001), with a SDEC of 22.9 h, a SDECV of 24.3 h, and SDEP of 23.2 h.

**Fig. 4:**
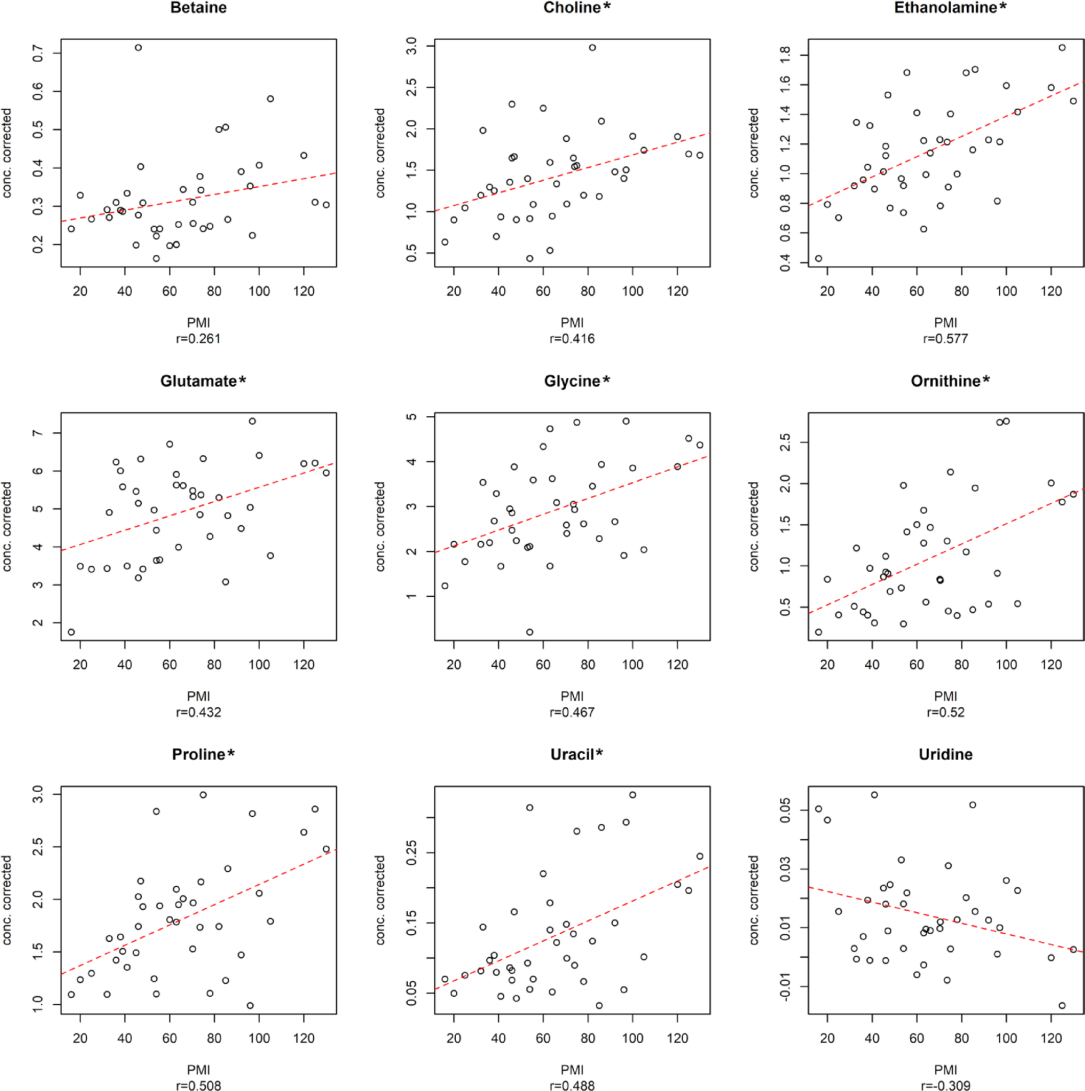
Relevant predictors for oCPLS2 model in 16–130 h PMI window. The dashed red line indicates the linear regression between the metabolite concentration corrected by age and the experimental PMI; r is the Pearson correlation coefficient. (*) indicated metabolites discovered by MLR analysis controlling the false discovery rate at level 0.05

As shown in Fig. 4, eight of the identified predictors exhibited a positive correlation with PMI (Betaine, Choline, Ethanolamine, Glutamate, Glycine, Ornithine, Proline, and Uracil) while one, namely Uridine, displayed a negative correlation as PMI increased. Notably, seven of these nine predictors were discovered by MLR analysis controlling the false discovery rate at level 0.05.

### Regression model on the PMI window 16–100 h

While the model showed significant enhancements in robustness and SDEP for PMIs up to 130 h, its performance remains relatively far from the proof-of-concept results on the model up to 100 h. This discrepancy could arise from the limited capacity of the proof-of-concept dataset to reflect interindividual variability or from the heightened complexity of biological processes linked to PMIs beyond a certain time in the post-mortem.

To address this, a third model was developed using a larger dataset, narrowing the time window to align with the proof-of-concept range of up to 100 h. A weak correlation between age and the investigated parameters was again observed (*r* = 0.23, *p* = 0.19), with no significant correlation with sex. The dataset was split into a training set of 35 samples and a test set of 15 samples, with both sets maintaining balanced distributions of PMI, sex, and age. An oCPLS2 model, utilizing stability selection and focusing on nine relevant predictors, was employed to examine the relationship between PMI and PF metabolome. This model identified one predictive score component, achieving an R^2^ of 0.332 (*p* = 0.007) and a Q^2^ of 0.156 (*p* = 0.003), along with a SDEC of 18.6 h, a SDECV of 20.4 h, and a SDEP of 16.7 h.

**Fig. 5:**
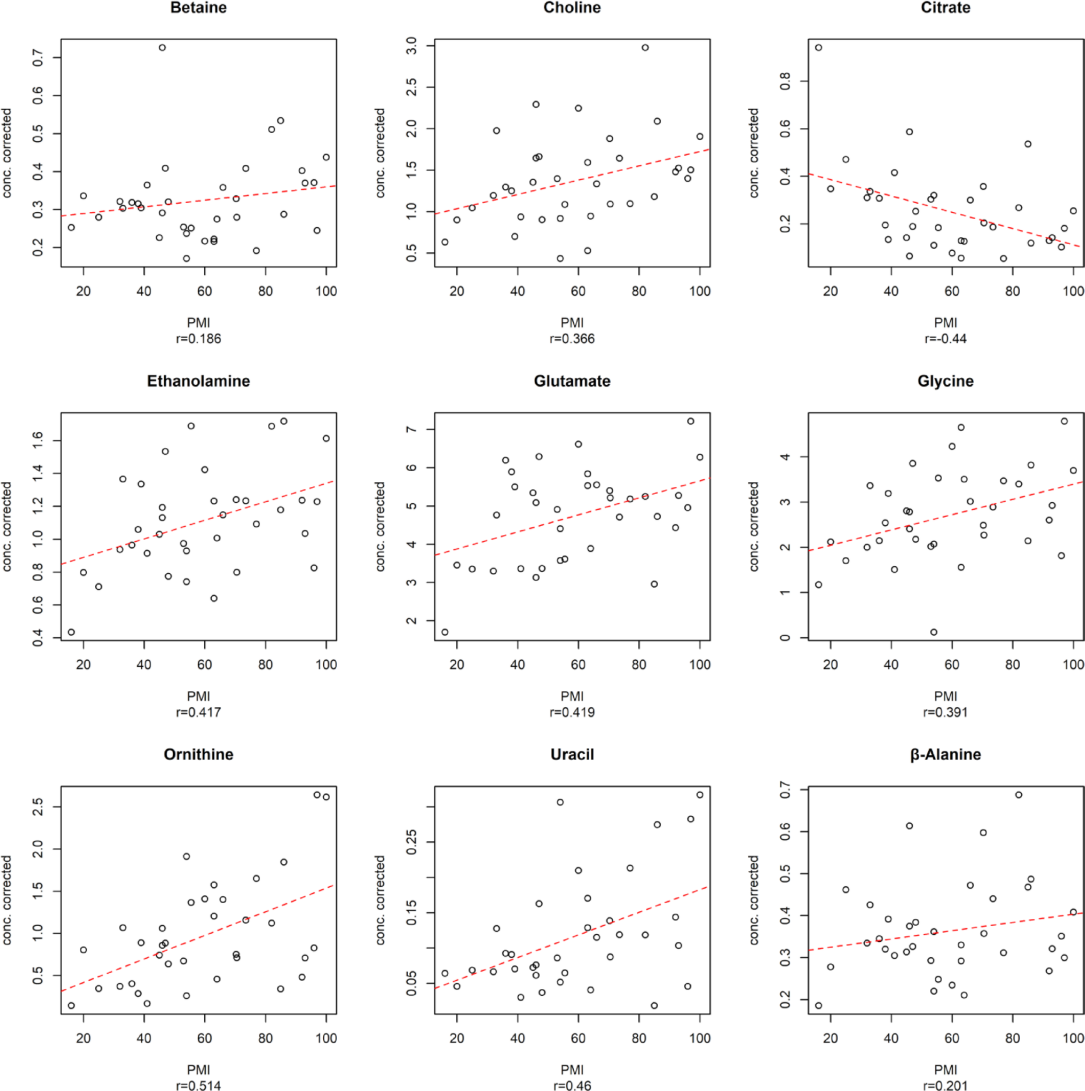
Relevant predictors for oCPLS2 model in 16–100 h PMI window. The dashed red line indicates the linear regression between the metabolite concentration corrected by age and the experimental PMI; r is the Pearson correlation coefficient

As illustrated in Fig. 5, nine predictors demonstrated a positive correlation with PMI (Betaine, Choline, Ethanolamine, Glutamate, Glycine, Ornithine, Uracil, and β-Alanine), whereas one metabolite, Citrate, showed a negative correlation with increasing PMI.

### Classification approach

To further explore PMI, a PLS-based classification model was employed within the narrowed 16–100 h time-window, utilizing the final dataset of 50 samples.

The PMI range was divided into two segments: less than 48 h (class ‘low’ *n* = 20) and greater than 48 h (class ‘high’ *n* = 30). An oCPLS2C model was developed to predict the PMI class based on the quantified 46 metabolites, focusing solely on relevant predictors. The samples were split into a training set (*n* = 28, n_low_ = n_high_ = 14) and a test set (*n* = 22, n_low_ = 6, n_high_ = 16). After autoscaling the data, the model revealed one component, with an MCC in calculation of 0.512 (*p* = 0.034), an in cross-validation equal to 0.433 (0.030), and an MCC of 0.418 when predicting the test set. The model successfully passed the randomization test (1000 random permutation) with α = 0.05.

Results from the calculation of the training set and the prediction of the test set are presented as confusion matrices in Table 3. Notably, when applied to the test set, misclassification errors were limited to the class ‘high’, where only about half of the samples were correctly predicted, while predictions for the class ‘low’ were consistently accurate. This suggests that this model excels at identifying ‘low’ PMIs, suggesting its potential as an effective screening tool for deaths occurring beyond 48 h.

**Table 3:** Classification model: confusion matrices obtained calculating the training set and predicting the test set

Training set			Test set		
Class	Calc. low	Calc. high	Class	Pred. low	Pred. high
Low	12	2	Low	6	0
High	2	12	High	9	7

**Fig. 6:**
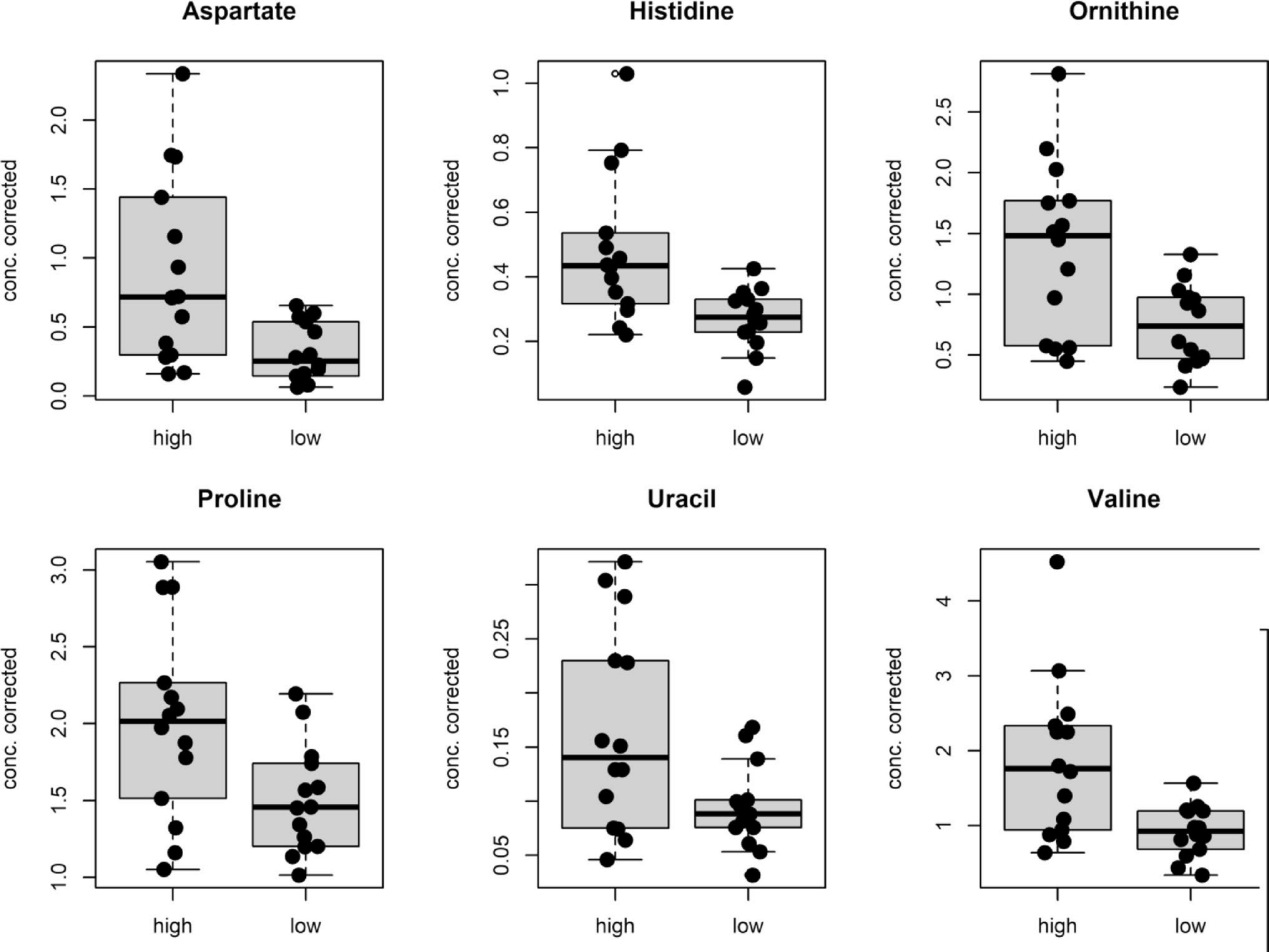
Boxplots of the relevant predictors discovered by PLS-analysis considering the 16–100 h PMI window. Black points are used to represent the metabolite concentration corrected by age of the training samples

Figure 6 presents the six relevant predictors identified in the classification model, namely aspartate, histidine, ornithine, proline, uracil, and valine. Notably, all the relevant predictors are significantly elevated in the class high indicating a potential biological significance as PMI increase.

**Fig. 7:**
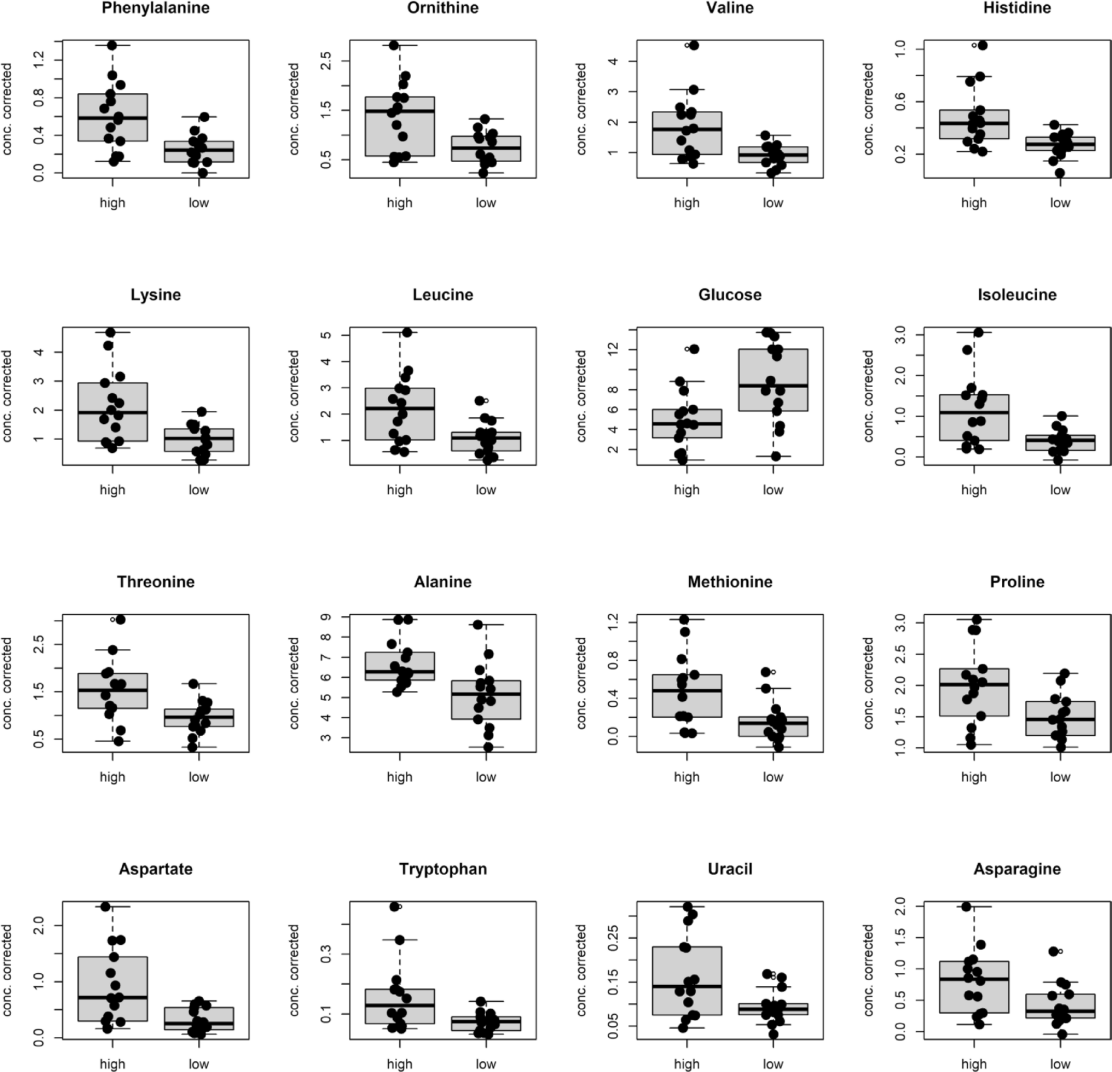
Boxplots of the predictors discovered by logistic regression analysis considering the 16–100 h PMI window. Black points are used to represent the metabolite concentration corrected by age of the training samples

Finally, Fig. 7 shows the relevant predictors selected through logistic regression analysis considering *p* < 0.05 for their regression coefficient. Among an expanded set of 16 relevant metabolites, that includes all the metabolites discovered by PLS-analysis, glucose is the only one metabolite decreased in the class high.

Remarkably, of these 16 relevant metabolites, 14 were amino acids, comprising 13 proteogenic amino acids and one, non-proteogenic amino acid, ornithine.

#### Hypoxanthine behavior

**Fig. 8:**
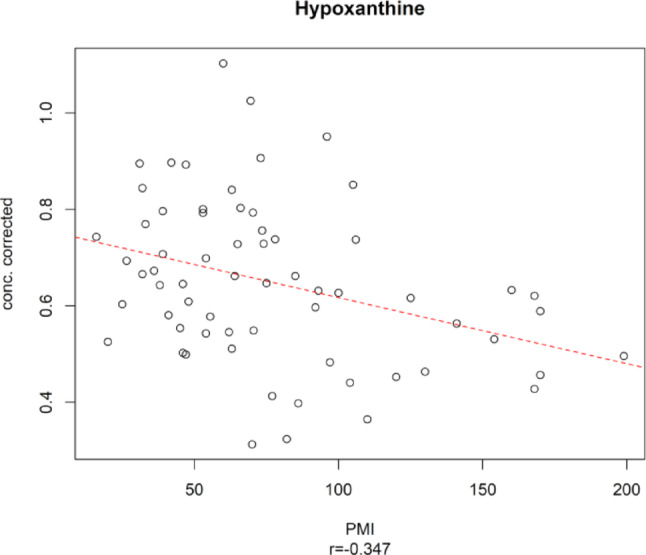
Concentration of Hypoxanthine vs. PMI. The dashed red line indicates the linear regression between the metabolite concentration corrected by age and the experimental PMI; r is the Pearson correlation coefficient

In the proof-of-concept study, hypoxanthine emerged as the sole significant metabolite exhibiting a negative correlation with PMI (Chighine et al., 2023). However, in the wider PMI time frame of the extended dataset (up to 199 h), it was not identified among the key metabolites, even though the negative trend persisted (Fig. 8).

Notably, when examining the metabolic pathway of Hypoxanthine - a well-known by-product of ATP degradation under hypoxic conditions – its precursor, Inosine, exhibits a consistent declining trend with PMI. Moreover, Inosine was among the relevant predictors in the PMI regression model within the 16–199 h time frame (see Fig. 3).

## Discussion

Before the proof-of-concept experiment (Chighine et al., 2023) little was known about the human PF metabolomic composition (Yang et al., 2023), especially in the post-mortem setting. Our first work on PF demonstrated that metabolomics composition of PF samples is maintained regardless the pre-analytical extraction protocol used, implying that the explored metabolome remains stable. Furthermore, since sampling was basically performed without exclusion criteria, the metabolomic composition did not appear related to sex and it was not possible to make any inference on the cause of death due to their diversity, although a moderate relationship between PMI and age was detected. In other terms, metabolomics differences in the metabolome within individuals due to different extraction protocols were negligeable compared to the inter-individual variability.

Although the proof-of-concept study offered critical insights into PF metabolomics, its conclusions were drawn from a limited number of samples, necessitating further confirmation of its reproducibility using a wider number of samples (Chighine et al., 2023).

In the present work we firstly assessed the goodness of the proof-of-concept results focusing on comparison between the metabolomic profiles of the same individuals obtained in two different extraction and ^1^H NMR analysis. While ^1^H NMR is widely acknowledged as a robust and highly reproducible analytical method (Emwas et al., 2019), we evaluated the experimental reproducibility on 23 samples from the proof-of-concept dataset (one sample having been fully utilized in prior tests) extracted with LLE protocol. Notable, despite the complex nature of the experiment - which involved multiple steps, from extraction procedure to pre- and post-processing of spectral data, 46 out of the 50 quantified metabolome exhibits exceptionally high similarity (≥ 90%) reflecting full consistency between the two independent experiments. Furthermore, this consistency translated into superposable performance in PMI prediction using data from the new batch data, underscoring robust reproducibility. Interestingly, within the time-window up to 100 h, models constructed selecting only the 46 metabolites with high similarity resulted in enhanced prediction accuracy.

Based on these observations, the proof-of-concept findings were demonstrated to be solid, comparable, and reproducible, hence supporting the expansion of the dataset to further explore PMI-related changes in PF metabolome.

Broadening the sample collection allowed for an investigation of the 100 h time-window using a dataset more than twice in size (*n* = 50) than the original. At the same time, this expansion introduced a markedly greater level of inter-individual variability, which could have potentially presented a significant obstacle to the proposed approach and explaining the reduced robustness observed in the model. Nevertheless, the prediction error across the full range shows only a modest increase from 13 to 16.7 h. Stated alternatively, despite the added complexity arising from the substantial rise in the inter-individual variability, the prediction ability of the oCPLS2 regression models remains largely unaffected.

Given the proof-of-concept experiment’s suboptimal performance for extended time-windows, the expanded dataset enabled a more thorough exploration of higher PMIs. This led to the development of a regression model based on 57 PF samples, spanning from 16 to 130 h, extending the experimental framework by more than a full day. Compared to the 100-hour model, this version exhibited higher R² and Q² values, indicating an improved ability to intercept the underlying metabolic processes. The model’s predictive performance was substantially comparable to that of the 100-hour model, with a prediction error of 23.2 h, showing an increase of 6.5 h over the additional 30-hour range. While the 16- to 100-hour time-window choice was driven by limitations of the proof-of-concept, the extension up to 130 h maximizes sample numerosity while preserving a high degree of PMI homogeneity in the present dataset.

Consistently with the proof-of-concept findings, models developed on samples with longer PMIs showed higher prediction errors, reaching 42.1 h across the 16-to-199 h range, suggesting a potential limitation for their use. The challenges associated with this time-window may stem from either the growing complexity of metabolomic processes (e.g., microbial co-metabolism) or the practical difficulties in securing samples with comparable PMIs, leading to a reduced sample size for these later intervals.

To frame these findings within forensic and legal setting, it is essential to acknowledge the significance of a method’s error and how such errors are evaluated in Court under the Daubert lens (Gelderman et al., 2021). From the experimental perspective, the ‘*known or potential rate of error*’ stands out as pivotal and perhaps the most rigorous element of the Daubert criteria. While the error rate serves as a key measure of a method’s reliability and performance, the Daubert standards do not mandate a specific error threshold for admissibility, rather it suffices that the rate is documented and understood. This is especially relevant for PMI estimation, which lacks reliable tools beyond 36–48 h post-mortem. In this scenario, even a method with a substantial error margin could mark a significant improvement over the absence of any method. The core tenets of the Daubert standards, particularly regarding the known or potential error rate, were integrated into the experimental design. Although the direct application of our results in a courtroom may remain a future prospect, it is vital to assess the prediction error rates of the regression models from a comparative and relative perspective.

When the errors of the regression models were assessed as percentages rather than absolute values, even those deemed underperforming exhibit relative error ranges comparable to those of the currently available methods. Of note, our models also shared with Henssge’s nomogram a worsening in prediction errors as the PMI window extends (Henssge, 1988). This perspective opens additional considerations of notable forensic importance. A substantial portion of the prediction error percentage appears to be surprisingly tied to the biological processes occurring in the initial phase of the time-window under investigation. In subsequent phases, where the overlap of exogenous metabolisms likely emerges, there is only a modest, albeit progressive, increase in error.

The interval between 100- and 130-hours post-mortem emerges as a compelling focus for research. During this later stage, not only does the model exhibit markedly improved robustness, but the percentage increase in prediction error also remains limited to just 1%, despite the time-window was expanded by more than 30% (see Table 4).

**Table 4:** Comprehensive report of prediction performance of regression models

Experiments	Samples	Window (h)	Error (h)	Error (%)
Proof-of-concept dataset	24	16–170	34	20
	20	16–100	13	13
Extended dataset	65	16–199	42.1	21.15
	57	16–130	23.1	17.7
	50	16–100	16.7	16.7

From a different point of view, it is essential to acknowledge that within the criminal justice system, analytical techniques are not primarily employed to corroborate hypotheses but more often to refute them which can significantly influence the resolution of an investigation or the verdict in a trial. This perspective led to the decision to perform a classification model, which exhibited high accuracy in detecting deaths occurred at PMI ≥ 48 h. A comprehensive analysis of the classification approach, combined with the regression model built on the 16–130 h window, suggests that PF metabolomics can serve as a reliable forensic screening tool for PMIs ≥ 48 h. This finding aligns with the inner anatomical position of PF, which likely minimizes metabolic changes during the early PMIs. Moreover, this suggests that PF metabolomics could be used as a complementary tool along with the Henssge’s Nomogram for estimating PMI in the first 48 h (Henssge, 1988; Henssge et al., 1988).

The dataset used in this work was underpowered for drawing conclusive inferences regarding metabolic pathways implicated. Despite this limitation, Choline, Ethanolamine, and Glycine identified as significantly correlated with PMI < 100 h in the proof-of-concept experiment – and previously detected also in our PMI estimation model based on sheep VH (Locci et al., 2023)– were confirmed among the relevant predictors for PMI estimation models up to 130 h after death. This highlights the post-mortem role of these metabolites in PF and in other biological matrices among human and animal models (Locci et al., 2021; Zelentsova et al., 2020; Musshoff et al., 2011; Michaelis et al., 1996; Chighine et al., 2025).

The only metabolite which was not confirmed as significantly correlated with PMI in this experiment was Hypoxanthine, a well-kown metabolite in forensic sciences indicating ATP consumption (Rognum et al., 2016). Interestingly, in the proof-of-concept study, Hypoxanthine stood out as the sole metabolite exhibiting a significant decreasing trend (Chighine et al., 2023) a result that contrasts with other findings. This peculiar observation was confirmed in the systematic review by Cardinale et al., which noted that our study was unique in reporting a decline in hypoxanthine levels during early PMIs (Cardinale et al., 2025).

Of note, hypoxanthine’s lack of significance in the current study may be due to its catabolism. This inference is supported by the negative correlation of its precursor, inosine, confirmed in the 199-hour model, where it ranked among the relevant predictors, while other intermediates of hypoxanthine metabolic pathways could not be identified by ^1^H NMR.

Moreover, when comparing proof-of-concept and extended dataset PMI estimation models on the 100 h PMI window, Citrate was confirmed among the significant metabolites correlated with PMI once the factor age is considered. Of note, Citrate, an intermediate of TCA cycle, showed a strong inverse correlation with PMI suggesting its depletion in the early post-mortem phase. Such behavior was already observer in bones on a significantly longer PMI window (Wilson et al., 2017).

Among the other metabolites, those implicated in the pyrimidine salvage pathway were found to be significantly correlated with PMI in our models (Chandel, 2021). The recurring presence of Uracil, a pyrimidine base, appears fascinating due to its relationship with the precursor Uridine, another indicator of ATP consumption (Harkness, 1988) which was found to be inversely related with PMI in our results. In conclusion, Uracil and Uridine relationship with b-Alanine deserves further investigation to elucidate PMI major implications in PF.

Beyond the comparison with the proof-of-concept experiment, the regression model for time-windows up to 100- and 130-hours post-mortem showed significant overlap among relevant predictors such as Betaine, Glutamate, Ornithine, and Uracil, emerging as consistent across the two models.

Conversely, the regression model constructed for the full time-window up to 199 h revealed distinct peculiarities, including a greater proportion of inversely correlated metabolites (Citrate, Glucose, and Inosine) and, intriguingly, the presence of Formate, an indicator of bacterial metabolism previously observed in the 170 h proof-of-concept model and shown to correlate with PMI in human blood specimens (Donaldson et al., 2013).

It is worth of note that, the nature of the relevant predictors in the classification model proposed significantly differs from those in the regression models, consisting predominantly of amino acids (14 out of 16 metabolites). This observation points to substantial post-mortem proteolysis occurring in the PF neighborhood, namely myocardium and parietal pericardium, consistently to previous observations on heart tissue (Zissler et al., 2020).

Prior research has identified a linear correlation between amino acids and PMI in human VH, specifically Taurine, Aspartate, Glutamate (Girela et al., 2008), and Tryptophan (Ansari et al., 2017). Despite the different biological matrix, Aspartate and Tryptophan emerged as significant metabolites in our classification model, both exhibiting elevated concentrations in the PMI class > 48 h. Notably, among the two other significant metabolites, Glucose aligned with the 16–199 h regression model, showing an inverse correlation with PMI.

The current study is subject to several limitations. Firstly, although expanding the sampling, the dataset is still relatively underpowered especially on longer PMI’s (beyond 130 h). This also influences the possibility of investigating different comorbidities and causes of death and their effect on the post-mortem metabolome. Secondly, PFs were obtained from medico-legal cases, where PMI was frequently estimated using circumstantial evidence or traditional thanatological indicators. Thirdly, bodies were refrigerated in morgues for varying periods prior to autopsy reasonably resulting in a slowdown of time-related metabolic processes and potentially underestimating predicted PMIs. Lastly, results were obtained from a single analytical platform, that, even if very robust, lacks sensitivity, hampering the detection of a wider range of metabolites, namely the lipophilic ones. A future implementation of the design of this study with a complementary analytical platform (i.e., LC-MS) able to detect the lipophilic share of the PF metabolome will allow to implement the number of relevant PMI predictors.

Despite these limitations, these findings indicate an optimal intra-laboratory reproducibility of the proposed methodology and moreover demonstrate that if the samples are well preserved during both storage and delivery the post-mortem metabolome is not affected by possible confounding factors such as collection in different institutes and by different pathologists. Furthermore, our results confirmed that in the PF metabolome, PMI is only moderately influenced by age while it is not affected by sex. Of great forensic interest, our results suggest that PF metabolomics could be potentially used in a critical time-window, namely around and beyond 100 h after death, when no tool for PMI estimation is currently available (Ruiz López et al., 2025; Singh et al., 2025).

In conclusion, the results of this work corroborated our previous results confirming the potential of PF as a biofluid of interest for post-mortem metabolomics. Hence, this proof-of-concept approach deserves to be translated on a wide dataset and investigated with different analytical platforms. Due to the peculiar physiopathology, PF metabolomics appears intriguing to investigate the cause and mechanism of death, unless modifications related to PMI are fully taken into account, either standardising the samples according to PMI or constraining the model to this factor.

## Supplementary Information

Below is the link to the electronic supplementary material.

Supplementary Material 1

## Data Availability

Datasets generated and/or analyzed during the current study are available from the corresponding author on reasonable request.
