# Supplementary material for: PMI estimation through 1H NMR metabolomics on human pericardial fluid: a validation study
Source: Metabolomics. 2025 Nov 15;21(6):174. doi: 10.1007/s11306-025-02376-3 (PMC12619721; doi:10.1007/s11306-025-02376-3)
Supplement: Supplementary file 1 — Supplementary Material 1 [file 11306_2025_2376_MOESM1_ESM.docx]

**PMI estimation through ^1^HNR metabolomics on human pericardial fluid: a validation study.**

Alberto Chighine^1,^ *, Matteo Stocchero^2^, Fabio De-Giorgio^3,4^, Riccardo Fratini^1^, Giorgia Fanunza^1^, Radhika Kesharwani^1^, Camilla Gozzelino^1^, Matteo Nioi^1^, Ernesto d’Aloja^1^, Emanuela Locci^1^

^1^Department of Medical Sciences and Public Health, Section of Legal Medicine, University of Cagliari, Cagliari, Italy.

^2^Department of Women’s and Children’s Health, University of Padova, Padova, Italy.

^3^Department of Health Surveillance and Bioethics, Section of Legal Medicine, Catholic University of Rome, Rome, Italy.

^4^Fondazione Policlinico Universitario A. Gemelli IRCCS, Rome, Italy.

**Supplementary Table 1**: Compounds identified by ^1^H NMR spectroscopy.

| **Compound** | **PubChem (CID)** | **Compound** | **PubChem (CID)** |
| --- | --- | --- | --- |
| 3-Hydroxybutyrate | 92135 | Lactate | 108689 |
| Acetate | 176 | Leucine | 6106 |
| Acetone | 180 | Lysine | 5962 |
| Alanine | 5950 | Maltose | 439186 |
| Asparagine | 6267 | Mannose | 18950 |
| Aspartate | 5960 | Methanol | 887 |
| Betaine | 248 | Methionine | 6137 |
| Butyrate | 264 | Nicotinurate | 68499 |
| Choline | 305 | Ornithine | 6262 |
| Citrate | 311 | Phenylalanine | 6140 |
| Creatine | 586 | Proline | 145742 |
| Creatinine | 588 | Propionate | 1032 |
| Dimethylamine | 674 | Serine | 5951 |
| Ethanolamine | 700 | Succinate | 1110 |
| Formate | 284 | Taurine | 1123 |
| Fumarate | 723 | Threonine | 6288 |
| Glucose | 5793 | Trimethylamine | 1146 |
| Glutamate | 33032 | Tryptophan | 6305 |
| Glutamine | 5961 | Tyrosine | 6057 |
| Glycerol | 753 | Uracil | 1174 |
| Glycine | 750 | Uridine | 6029 |
| Histidine | 6274 | Valine | 6287 |
| Hypoxanthine | 790 | myo-Inositol | 892 |
| Inosine | 6021 | sn-Glycero-3-phosphocholine | 439285 |
| Isoleucine | 6306 | β-Alanine | 239 |
